# Sexual Offspring Production by Acorn Ant *Temnothorax crassispinus* Colonies Is Associated with the Colony Size but Not with the Volume of the Nest Cavity

**DOI:** 10.3390/ani15010049

**Published:** 2024-12-28

**Authors:** Mateusz Rolski, Anna Gruszka, Mariia Marczak, Sławomir Mitrus

**Affiliations:** Institute of Biology, University of Opole, Oleska 22, 45-052 Opole, Poland; matirol@op.pl (M.R.); ania4504@gmail.com (A.G.); marczakmam@gmail.com (M.M.)

**Keywords:** cavity nesting ants, energy allocation, production of sexual individuals, per capita, colony size

## Abstract

Nest sites are important for ants, as the nests provide refuge against enemies and ensure optimal conditions for the brood development. As the construction of a nest is a time and energy consuming activity, many ant species dwell in ready-for-use cavities. For them, choosing a good nest site is important, as the quality of nest site could affect factors such as the energy allocation and production of sexual individuals. Ants of genus *Temnothorax* inhabit cavities in, e.g., acorns and twigs, and these ant colonies prefer larger cavities as nest sites. It was previously shown that colonies inhabiting more durable nest sites invested more resources in sexual production. The aim of this study was to find out whether the volume of a nest cavity influences the sexual offspring production of acorn ant *Temnothorax crassispinus* colonies. For this purpose, a three-month long laboratory experiment was performed. Colonies of the *Temnothorax* ant were kept in artificial nest cavities differing in volume. It was found that there was no correlation between the productivity per capita and the colony size. More populous colonies produced more sexual individuals; however, we found no statistical difference in the production of sexual individuals between the colonies that inhabited cavities of different volumes.

## Simple Summary

Ants are highly abundant animals in almost all terrestrial ecosystems. For ants, nest sites are important, as the nest protects them against predators and can ensure optimal conditions for brood development. Additionally, the quality of a nest site can, for example, affect the production of a colony. Many ant species live in small colonies and do not construct nests, but instead dwell in available cavities, for example inside seeds or twigs. Modifying such cavities is difficult or in many cases impossible, thus cavity volume is important for colonies. We studied acorn ants which typically inhabit empty acorns. Generally, acorn ants prefer larger cavities. The aim of this study was to find out whether the volume of a nest cavity influences the production of new queens and males. For this purpose, during a three-month long laboratory experiment, we kept ant colonies inside artificial nest cavities differing in volume. Larger colonies produced more new queens and males; however, we found no difference in the production of sexual individuals between the colonies that inhabited cavities of different volumes.

## 1. Introduction

Nest sites are an important part of the ecology of social insects, including ants. For ants, nests provide a refuge from predators, as well as protecting them and their brood from extreme environmental factors, like low temperatures, ensuring optimal conditions for brood development and influencing the ants’ social interactions [1,2,3]. Many ant species actively excavate nest cavities, but this is a time and energy consuming activity [4]. Thus, colonies of other species—called ‘cavity nesting ants’—dwell in ready-for-use spaces, such as cavities in wood and seeds or spaces between rocks [1]. For cavity nesting ants, the parameters of potential nest site, such as the volume of the cavity and size of the hole which can be used as an entrance, are important factors that can affect the structure of the ant communities, as wider diversity of holes and the volume of cavities allows more species to use such available sites (e.g., [5,6,7]). Cavity nesting ants can modify the potential nest sites; for example, a modification of the entrance hole is a common behaviour in ants [8,9,10]. Modifying the cavity volume inside seeds or twigs is difficult or in many cases impossible; however, for example, it was reported that *Temnothorax nylanderi* can enlarge the cavity [11] and that *Leptothorax acervorum* builds loose partitions from wood fragments inside the cavity [12]. Nevertheless, larger changes in the volumes of the cavities in seeds or wood used as nest sites are difficult for ant colonies. Thus, the cavity volume inside a potential nest site is important for the ant colony.

An example of cavity nesting ants are those of the genus *Temnothorax*. Depending on the species, they inhabit cavities in the dry branches of living trees, rocks, fallen twigs and seeds or in the spaces under stones [13,14]. Under natural conditions, good nesting sites for these cavity nesting ants are limited resources [1,5,11,15] and, in ants of the genus *Temnothorax*, there is intense competition for nest sites [11,16,17]. Nesting sites, such as cavities in seeds and twigs, are also ephemeral, e.g., they can be accidentally crushed or no longer inhabitable as a result of decaying processes [18]. Thus, the colonies can be forced to move frequently to new sites, up to several times per season [16,19]. Such migrations are both costly and dangerous; therefore, choosing a good nest site is crucial for cavity nesting ant colonies. Additionally, the quality of the inhabited nest site can influence reproduction; for example, it was shown that ant colonies dwelling in inferior sites, like empty acorns, grass stems or rolled up leaves (compared to those dwelling in sturdy nest sites, like durable sticks), have a more male-biased sex allocation ratio (i.e., invest more energy in the production of males than females) [20].

When offering cavities with volumes similar to these found for specific species under natural conditions, *Temnothorax* ant colonies prefer larger ones (i.e., cavities having a larger volume) [21,22]. During a field experiment, *T. crassispinus* ant colonies more frequently inhabited larger artificial cavities [23]. In addition, the colonies that inhabited larger nest cavities invested more in sexual individuals [23]; however, as colonies that inhabited larger nest cavities were simultaneously more populous, it was not possible to determine whether the effect (i.e., that colonies invested more in sexual individuals) was due to the colony size or the volume of the cavity. In another study involving *T. nylanderi* ant colonies, it was shown that the colonies inhabiting sturdy nest sites, like durable sticks, invested more resources in the production of sexual individuals [24]. The aim of this study was to find out whether the volume of a nest cavity influences the sexual offspring production of acorn ant *T. crassispinus* colonies. To this end, we performed a laboratory experiment during which colonies of the ant were kept in artificial nest cavities differing in volume.

## 2. Materials and Methods

In this study, acorn ant *Temnothorax crassispinus* colonies were used. This ant species is present throughout Central and Eastern Europe. The colonies are small, typically ranging from a few dozen to about 200 workers. They dwell mostly in cavities in old acorns and small, fallen twigs [13,25,26], and such cavities are impossible or difficult to enlarge.

On 13 April 2023, near Opole, Poland (GPS 50.638151, 18.125039), we collected acorns containing ant colonies, i.e., ants with broods in different stages of development. In the field, we put each collected acorn in a separate plastic box and transported these to a laboratory. In the laboratory, we opened the acorns, then captured the individuals with an aspirator and counted the ants. We collected 90 colonies: 30 queenless (13–167 workers, mean = 65.3, median = 58, standard error SE = 8.3); 56 containing one queen (6–228 workers, mean = 100.4, median = 68.5, SE = 7.7); three containing two queens each (with 88, 141 and 179 workers, respectively); and one colony with three queens and 122 workers.

We chose 48 colonies containing 28–228 workers (mean = 110.6, median = 103.5, SE = 7.7) with one queen and brood (cf. [27]). We randomly divided the colonies into three experimental groups, 16 colonies to each of the three groups (see Table 1). Then, each colony was transferred to a square Petri dish (10.2 cm × 10.2 cm × 1.9 cm) having a thin plaster base, with an artificial nest site on top. Such a nest site was a cavity between a piece of cardboard and a microscope slide (or half a microscope slide), separated by a plexiglass frame (3 mm thick) and covered with a piece of red translucent filter (the design of such nest sites is presented, e.g., in [28]). All the cavities of the artificial nest sites used in the study had the same entrance sizes and were of the same shape: the cavities of the three experimental groups differed only in the cavity length, and hence in the volume of the nest (cf. [23], for the shapes of the plexiglass frames). The volumes of the nest cavities used in the study were c.a. 470, 860 and 1760 mm^3^ for the ‘small’, ‘medium’ and ‘large’ nests, respectively—such volumes corresponded in size to the cavities inside empty acorns in which the ants of the genus *Temnothorax* dwell (cf. [21]).

During the acclimatisation period (14 days) none to five (median = 1) workers per colony died. Four colonies did not inhabit the artificial nest sites, i.e., the colonies with 33–228 workers, all from the ‘small’ cavity group. The other four colonies (containing 149–188 workers), which were also placed in Petri dishes with ‘small’ cavities, firstly inhabited the nest sites, but later could not fit in these cavities, and part of the workers and brood members stayed outside, near the nest entrance. Additionally, in one colony in a ‘large’ nest cavity, the queen died. In the end, only a small number of colonies from the ‘small’ cavity group remained in the experiment (see Table 1).

The dishes with the ant colonies were kept in a thermostatic cabinet where a daily cycle of day/night was maintained, with a regime of 12 h/12 h at temperatures of 20 °C/10 °C, respectively, until 24 June; from that day onwards, the regime was shifted to 14 h/10 h at 27 °C/17 °C (the same as the artificial spring and summer conditions previously used in experiments on *T. nylanderi* (e.g., [24], with conditions that are in accordance with the field conditions during the respective seasons of the year in central Europe). The ants were fed twice a week, alternately with frozen fruit flies *Drosophila hydei* and honey, or using c.a. 3 mm × 3 mm × 3 mm of jelly-like food prepared according to the Bhatkar diet [2]. The ants were provided with *ad libitum* water. During the feeding, dead workers were collected and counted. After three months, on 27 July, the final number of workers (including pupae of workers) in each colony and the number of sexual individuals (including the pupae of sexual individuals) produced by each colony were counted.

### Statistical Analyses

In order to estimate the cost of the production of sexual individuals, we adopted data from the literature for the ant *T. nylanderi* (a sibling species of *T. crassispinus*). According to the data, the dry mass, and hence the cost, of a gyne (a ‘young queen’) being produced is 3.02 times higher than that of a male, and the dry mass of a worker does not differ from the male’s dry mass [24,29]. Thus, the cost of production of a gyne by a colony was calculated as 3.02 × cost of the production of a worker or a male. We used three parameters of productivity: ‘the cost of production of sexual individuals’ (i.e., number of produced young gynes × 3.02 + number of produced males), ‘the colony productivity’ (i.e., ‘the cost of production of sexual individuals’ + number of new workers), and ‘the colony productivity per capita’ (i.e., ‘the colony productivity’ divided by the initial number of workers).

Pearson correlations were used to assess the relationship between the colony size (i.e., initial number of workers) and the colony productivity per capita. The general linear model (GLM) was used to determine whether the cost of production of sexual individuals was impacted by the cavity volume (i.e., ‘small’, ‘medium’ and ‘large’). The GLM model included the initial number of workers in a colony (number of workers at the beginning of the experiment) and the colony’s growth (increase in the number of new workers) as continuous predictors. All data sets were tested for normal distribution using the Shapiro–Wilk test. Skewed data were then transformed prior to the analyses: the data on the cost of production of sexual individuals were transformed before the analysis using cube root transformation for this purpose. All the statistical analyses were conducted using the software package Statistica, ver. 13 [30]. All of the probability values shown are two-tailed.

## 3. Results

Colonies in the ‘small’ cavities produced 0–52 (mean = 23.9, median = 23, SE = 5.8, *N* = 8) males; none of these colonies produced a gyne. Colonies in the ‘medium’ cavities produced 0–93 (mean = 19.5, median = 16, SE = 5.9, *N* = 16) males and 0–24 gynes (mean = 2.1, median = 0, SE = 1.6, *N* = 16). Specifically, 12 colonies produced males only, while two colonies produced gynes only, one colony produced both males and gynes, and one colony produced no sexual individuals. Colonies in the ‘large’ cavities produced 0–70 (mean = 19.0, median = 0, SE = 5.7, *N* = 15) males and 0–2 (mean = 0.4, median = 0, SE = 0.2, *N* = 15) gynes. Specifically, 11 colonies produced males only, while three colonies produced gynes only, and one colony produced both males and gynes.

Each colony produced new workers. Colonies in the ‘small’ cavities produced 56–229 workers (mean = 136.6, median = 117.5, SE = 19.9, *N* = 8), colonies in the ‘medium’ cavities produced 13–243 workers (mean = 116.5, median = 120, SE = 17.3, *N* = 16), and colonies in the ‘large’ cavities produced 25–256 workers (mean = 134.7, median = 141, SE = 16.0, *N* = 15).

**Figure 1:**
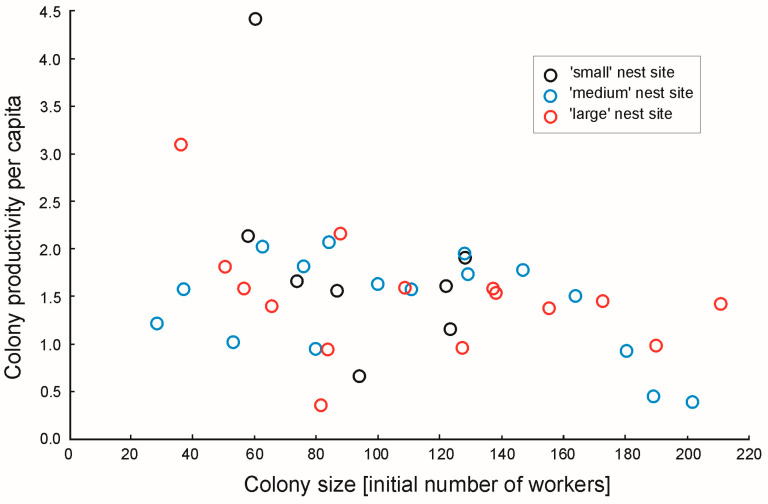
Colony productivity per capita in ant colonies of *Temnothorax crassispinus* in relation to the colony size. During the three-month long laboratory experiment, the colonies were kept in small (volume 470 mm^3^, *N* = 8), ‘medium’ (volume 860 mm^3^, *N* = 16) or ‘large’ (volume 1760 mm^3^, *N* = 15) nest cavities. There were no correlations between the colony productivity per capita and the colony size, in either of the three groups.

There was no correlation between the colony size (i.e., the initial number of workers) and the colony productivity per capita (*r* = −0.52, *N* = 8, *p* = 0.19, *r* = −0.44, *N* = 16, *p* = 0.089 and *r* = −0.36, *N* = 15, *p* = 0.18, for the ‘small’, ‘medium’ and ‘large’ nest cavities, respectively) (Figure 1).

**Figure 2:**
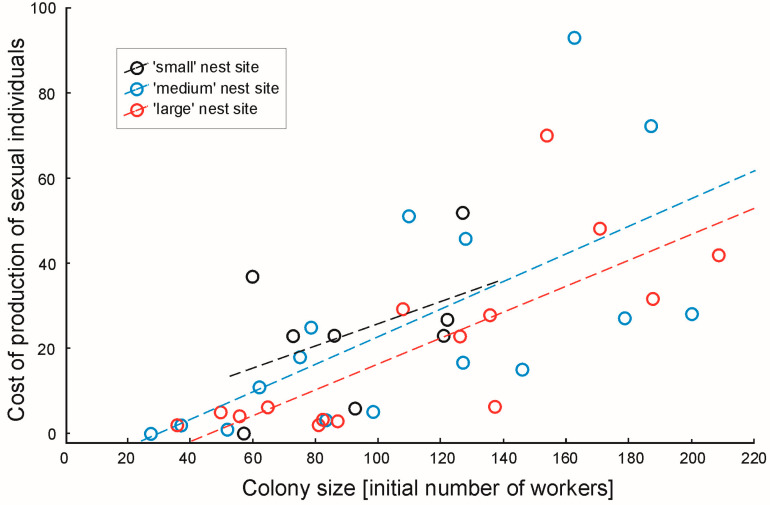
The cost of production of sexual individuals in ant colonies of *Temnothorax crassispinus* in relation to the colony size. During the three-month long laboratory experiment, the colonies were kept in ‘small’, ‘medium’ and ‘large’ nest cavities. The cost of production of sexual individuals was affected by the colony size (GLM, *p* < 0.0001), but there was no difference between the three groups (*p* = 0.37). In the figure, the raw data is presented; however, for the analyses the transferred data were used (see text for details).

There was no difference in the cost of production of sexual individuals between colonies inhabiting cavities of different volumes (GLM: F_2,34_ = 1.01, *p* = 0.37). The production of new workers did not affect the cost of production of sexual individuals (F_1,34_ = 0.26, *p* = 0.61); however, the initial number of workers did significantly affect the cost of production of sexual individuals, with more populous colonies investing more in the production of sexual individuals (F_1,34_ = 19.09, *p* < 0.0001) (see Figure 2).

## 4. Discussion

In this experiment, the more populous colonies invested more in production of sexual individuals, but we found no effect of the nest cavity volume on this production. It is generally believed that colony productivity per capita declines in more populous ant colonies, and in species with small colonies (cf. [31]), but such a relation creates Michener’s paradox: how can natural selection maintain social behaviour when it favours the evolution of smaller colony size (of course, in the absence of benefits to large colonies, like, e.g., higher survival rate) [32]. However, the hypothesis of decreasing efficiency with increasing colony size was not supported by the latest studies (cf. [32]) and, for the *Temnothorax crassispinus* ant, no decrease in colony productivity per capita was shown with increasing colony size [31]. Similarly, during this study, there was no correlation found between colony size and colony productivity per capita; however, these results should be interpreted with caution, as a post hoc power analysis (conducted with G*Power 3.1.9.7 [33]), using a value of the effect size of 0.5, showed that the power is low: 0.58 and 0.54 for the ‘medium’ and ‘large’ nest cavities, respectively, and only 0.28 for the ‘small’ nest cavities group.

Under natural conditions, there are many factors which could have an influence on ant colonies, and thus their energy allocation. For example, it was shown that colonies in ephemeral nest sites produce a more male-biased sex allocation ratio [20], and that food supply can affect the production of sexual individuals [34]. Due to the many factors which can affect the energy allocation of these insects, we performed our laboratory experiment. In particular, we wanted to check the effect of one factor—the volume of the nest cavity—on the production of sexual individuals. However, we additionally used colonies that were different in size: queenright colonies with 27–204 workers. Such differences in size are natural for *Temnothorax* ant colonies (see, for example, [25]), thus, we decided not to standardise the sizes of colonies before the start of an experiment.

Cavity nesting ants generally prefer larger cavities [21]. It has also been shown that the cavity volume in acorns inhabited by *T. curvispinosus* colonies is correlated with the colony weight [21], and for *T. nylanderi* a correlation was found between sites containing nest cavities and the number of workers in a colony [11]. However, the observed relationship between ant colony size and nest cavity volume could be a result of the nest site selection process (i.e., more populous colonies choose larger cavities), or could indicate that the volume of the nest cavity affects the colony’s growth (colonies dwelling in larger cavities produce more workers and thus, ultimately, they become more populous). Additionally, as colonies of acorn ants of the genus *Temnothorax* can frequently change nest sites (see the Introduction), data on colony sizes and cavity volumes collected under natural conditions may not be enough to explain the influence of cavity volume on energy allocation, as the field colonies collected may have changed their nest sites shortly before the collection.

During this study, only a part of the ‘small’ cavities was dwelled in by ant colonies. The volume of the potential nest site must be large enough to house the whole ant colony [21], and the ‘small’ cavities were too small for the more populous ant colonies. Nevertheless, even several less populous colonies did not dwell in the ‘small’ cavities. This, therefore, suggests that volume of cavity is important for ant colonies. Choosing larger sites would prevent space limitations on colony growth in the future, so colonies do not accept too small cavities [21]. In addition, small cavities may influence nest homeostasis (e.g., gas concentration), but could also indirectly influence the colony’s social organization through the effects of the spatial distribution of the ants and brood within the nest [21,35]. Ants are able to estimate the area of a potential nest site (cf. [36]); however, it could be more difficult to predict whether a potential site is sufficient for certain colony—this would require information about the size of the site, the number of workers and brood items in the colony, and a comparison of both sets of information. Therefore, if a site seems small, the colony may not settle in it (since such activity takes time and energy) but would look for other possible sites.

Colonies from ‘small’ cavities produced males only, and it was previously shown that colonies in ephemeral nest sites produce a more male-biased sex allocation ratio [20]. This could suggest that ‘small’ cavities were perceived as inferior; however, data are available for only eight colonies (see Materials and Method section). Nevertheless, it is important that most colonies dwelling in such small cavities produced sexual offspring, so colonies dwelling in such nest sites are able to contribute to the next generation, at least by providing males.

The cavity volumes used in the study (i.e., 470, 860 and 1760 mm^3^) correspond to the volumes of cavities naturally occupied by acorn ants (cf. [21]); however, the ‘small’ (i.e., 470 mm^3^) cavities were too small for more populous colonies (see the Materials and Methods section). During another laboratory experiment, it was shown that *T. curvispinosus* ant colonies selected larger cavities, i.e., 1.93 versus 0.76 mL [21]—these volumes are similar to the ‘medium’ and ‘large’ ones used during this study. An effect of the colony size on the cost of production of sexual individuals was found, with more populous colonies investing more in sexual individuals; however, we found no difference in the cost of production of sexual individuals between colonies kept in cavities of different volumes. As, during this study, most of the colonies produced males only, it was not possible to analyze the sex ratio.

## 5. Conclusions

Nest sites are important for ants, and it is known that the quality of a nest site affects the colony productivity (see above). For cavity nesting ants, the cavity volume of a potential nest site is an important factor. Firstly, the volume must be large enough to house the whole ant colony, and in this study the ‘small’ cavities were too small for the more populous ant colonies. Additionally, as cavity nesting ants generally prefer to occupy larger cavities, volume should be an important factor for them. In this study, no effect of the volume on the cost of production of sexual individuals was found; however, a colony of larger volume may influence other parameters that are important for ants, such as the possibility of the regulation of temperature or humidity (see [21]). Nevertheless, in this study, the Petri dishes with the artificial nest sites were kept in a stable condition.

## Figures and Tables

**Table 1:** Number of workers in *Temnothorax crassispinus* ant colonies used during this study. During the three-month long laboratory experiment, the colonies were kept in ‘small’, ‘medium’ and ‘large’ nest cavities (see text for details). Initially, 48 queenright colonies were randomly divided into three experimental groups; however, during the acclimatisation period some colonies did not inhabit the artificial nest sites; others firstly inhabited the nest sites, but later could not fit in these cavities, or a queen died. Thus, initial sizes, as well as sizes after the acclimatisation period (i.e., for which results were analysed) are shown. *N* is number of colonies, SE—standard error.

	Mean (SE)	Median	Min–Max
Colonies chosen to experiment			
the ‘small’ nest cavity group [*N* = 16]	112.3 (14.1)	107	33–228
the ‘medium’ nest cavity group [*N* = 16]	109.6 (13.6)	104.5	28–200
the ‘large’ nest cavity group [*N* = 16]	109.9 (13.0)	97.5	36–209
Colonies after acclimatisation period			
the ‘small’ nest cavity group [*N* = 8]	91.0 (9.8)	87	57–126
the ‘medium’ nest cavity group [*N* = 16]	108.7 (13.5)	104	27–200
the ‘large’ nest cavity group [*N* = 15]	110.6 (13.4)	107	36–204

## Data Availability

The original contributions presented in this study are included in this article/Supplementary Material; further inquiries can be directed to the corresponding authors.

## Supplementary Materials

The following supporting information can be downloaded at: https://www.mdpi.com/article/10.3390/ani15010049/s1, dataset and Table S1. Complementary metrics on colonies productivity.
