# Supplementary material for: Sexual Offspring Production by Acorn Ant Temnothorax crassispinus Colonies Is Associated with the Colony Size but Not with the Volume of the Nest Cavity
Source: Animals (Basel). 2024 Dec 28;15(1):49. doi: 10.3390/ani15010049 (PMC11718874; doi:10.3390/ani15010049)
Supplement: Supplementary file 1 [file animals-15-00049-s001.zip › animals_table-S1.pdf]

Sexual Offspring Production by Acorn Ant *Temnothorax crassispinus* Colonies Is Associated with the Colony Size but Not with the Volume of the Nest Cavity

Mateusz Rolski, Anna Gruszka, Mariia Marczak and Sławomir Mitrus

Supplementary material:

Table S1. Complementary metrics on colonies productivity. During the three-month long laboratory experiment, *Temnothorax crassispinus* colonies were kept in 'small', 'medium' and 'large' nest cavities (see text for details). Number of sexual offspring – absolute number of produced gynes and males by specific colony; worker to offspring ratio – number of workers on the end of the experiment to number of sexual offspring; sexual investment ratio total reproductive effort invested in sexual offspring relative to worker production; sexual offspring per unit of cavity volume; caste ratio – number of gynes to number of all sexual individuals produced by colony. *N* – number of colonies, SE – standard error.

	Mean (SE)	Median	Min–Max
**Number of sexual offspring**			
the 'small' nest cavity group [$N = 8$]	23.9 (5.8)	23	0–52
the 'medium' nest cavity group [$N = 16$]	21.6 (5.8)	17.5	0–93
the 'large' nest cavity group [$N = 15$]	19.4 (5.6)	4	1–70
Kruscall–Wallis test: $H_{2,39} = 0.37$, $p = 0.83$			
**Worker to offspring ratio**			
the 'small' nest cavity group [$N = 8$]	0.117 (0.024)	0.13	0.00–0.20
the 'medium' nest cavity group [$N = 16$]	0.101 (0.025)	0.09	0.00–0.37
the 'large' nest cavity group [$N = 15$]	0.075 (0.019)	0.03	0.01–0.27
Kruscall–Wallis test: $H_{2,39} = 2.16$, $p = 0.34$			
**Sexual investment ratio**			
the 'small' nest cavity group [$N = 8$]	0.169 (0.031)	0.18	0.00–0.27
the 'medium' nest cavity group [$N = 16$]	0.540 (0.340)	0.12	0.00–5.58
the 'large' nest cavity group [$N = 15$]	0.138 (0.033)	0.08	0.02–0.50
Kruscall–Wallis test: $H_{2,39} = 0.88$, $p = 0.65$			
**Sexual offspring per unit of cavity volume**			
the 'small' nest cavity group [$N = 8$]	0.051 (0.013)	0.05	0.00–0.11
the 'medium' nest cavity group [$N = 16$]	0.030 (0.008)	0.02	0.00–0.11
the 'large' nest cavity group [$N = 15$]	0.012 (0.003)	0.00	0.00–0.04
Kruscall–Wallis test: $H_{2,39} = 7.12$, $p = 0.029$			
**Sex ratio**			
the 'small' nest cavity group [$N = 7$]	0.000 (0.000)	0.00	0.00–0.00
the 'medium' nest cavity group [$N = 15$]	0.152 (0.091)	0.00	0.00–1.00
the 'large' nest cavity group [$N = 15$]	0.222 (0.106)	0.00	0.00–1.00
Kruscall–Wallis test: $H_{2,37} = 2.20$, $p = 0.33$			
